# Can Total Thyroidectomy Be Safely Performed by Residents? A Comparative Retrospective Multicenter Study

**DOI:** 10.1097/MD.0000000000003241

**Published:** 2016-04-08

**Authors:** Angela Gurrado, Rocco Bellantone, Giuseppe Cavallaro, Marilisa Citton, Vasilis Constantinides, Giovanni Conzo, Giovanna Di Meo, Giovanni Docimo, Ilaria Fabiola Franco, Maurizio Iacobone, Celestino Pio Lombardi, Gabriele Materazzi, Michele Minuto, Fausto Palazzo, Alessandro Pasculli, Marco Raffaelli, Frederic Sebag, Salvatore Tolone, Paolo Miccoli, Mario Testini

**Affiliations:** From the Department of Biomedical Sciences and Human Oncology (AG, GDM, AP, MT), University Medical School of Bari, Bari; Department of Surgery (RB, CPL, MR), University Medical School “Cattolica del Sacro Cuore,” Rome; Department of Medical and Surgical Sciences and Biotechnologies (GC), University Medical School “La Sapienza,” Rome; Department of Surgery (MC, MI), Oncology and Gastroenterology, University of Padova, Padova; Department of Anesthesiology (GC, GD, ST), Surgical and Emergency Sciences, Second University of Naples, Naples; Department of Surgical (GM, PM), Medical, Molecular Pathology, Critical Area, University Medical School of Pisa, Pisa; Department of Surgical Sciences (MM), University Medical School of Genoa, Genoa, Italy; Department of Thyroid and Endocrine Surgery (VC, FP), Imperial College London, London, UK; and Department of General and Endocrine Surgery (IFF, FS), Hôpital de la Timone, Marseille, France.

## Abstract

This retrospective comparative multicenter study aims to analyze the impact on patient outcomes of total thyroidectomy (TT) performed by resident surgeons (RS) with close supervision and assistance of attending surgeons (AS).

All patients who underwent TT between 2009 and 2013 in 10 Units of endocrine surgery (8 in Italy, 1 in France, and 1 in UK) were evaluated. Demographic data, preoperative diagnosis, extension of goiter, type of surgical access, surgical approach, operative time, use and duration of drain, length of hospitalization, histology, and postoperative complications were recorded. Patients were divided into 3 groups: A, when treated by an AS assisted by an RS; B and C, when treated by a junior and a senior RS, respectively, assisted by an AS.

The 8908 patients (mean age 51.1 ± 13.6 years), with 6602 (74.1%) females were enrolled. Group A counted 7092 (79.6%) patients, Group B 261 (2.9%) and Group C 1555 (17.5%). Operative time was significantly greater (*P* < 0.001) in B (101.3 ± 43.0 min) vs A (71.8 ± 27.6 min) and C (81.2 ± 29.9 min). Duration of drain was significantly lower (*P* < 0.001) in A (47.4 ± 13.2 h) vs C (56.4 ± 16.5 h), and in B (42.8 ± 14.9 h) vs A and C. Length of hospitalization was significantly longer (*P* < 0.001) in C (3.8 ± 1.8 days) vs B (2.4 ± 1.0 days) and A (2.6 ± 1.5 days). No mortality occurred. Overall postoperative morbidity was 22.3%: it was significantly higher in B vs A (29.5% vs 22.3%; odds ratio [OR] 1.46, 95% confidence interval [CI] 1.11–1.92, *P* = 0.006) and C (21.3%; OR 1.55, 95% CI 1.15–2.07, *P* = 0.003). No differences were found for recurrent laryngeal nerve palsy, hypoparathyroidism, hemorrhage, and wound infection. The adjusted ORs in multivariate analysis showed that overall morbidity remained significantly associated with Group B vs A (OR 1.48, 95% CI 1.12–1.96, *P* = 0.005) and vs C (OR 1.60, 95% CI 1.19–2.17, *P* = 0.002), while no difference was observed in Group A vs B + C.

TT can be safely performed by residents correctly supervised. Innovative gradual training in dedicated high-volume hospitals should be proposed in order to allow adequate autonomy for the RS and safeguard patient outcome.

## INTRODUCTION

During the last decades, hand in hand with the rise of the incidence of thyroid disease, the number of thyroidectomies has increased,^1^ thanks to the improvement of people screening and imaging techniques refinement.

Despite the raised demand for the surgical management of thyroid diseases, some studies have demonstrated that the operations are often performed by surgeons whose practice is not primarily focused on endocrine surgery.^1,2^ Indeed, the analysis of data between 1988 and 2000 from the National Inpatient Sample revealed that surgeons whose practice was made up of <25% of endocrine procedures performed 82% of thyroidectomies.^2^ On the other hand, several studies^3,4^ demonstrated the association between higher surgeon volume and better patient outcomes in all fields of surgery, and also in endocrine surgery.^1,5–7^ Maryland's reported data suggest that surgeons who have performed 100 or more thyroidectomies showed the lowest rate of complications, but the hospital volume is not statistically associated with the outcomes.^5,8,9^ To find the right balance between the patient safety and the need to train new surgeons with specific competences in endocrine surgery, remains the fundamental dilemma. If the peculiarities of a surgeon are a sufficiently high level of knowledge and the ability to perform a specific operation independently, the end point of a residency program should be represented by completion of an educational experience and by obtainment of appropriate technical skills.

No standardized criteria exist for declaring competence in thyroid surgery for residents, but some dedicated endocrine surgical programs on general surgery training have been introduced in order to foster resident competence in the surgical management of thyroid diseases.^10–14^ The introduction of these specific endocrine surgery programs seems to improve the quality of resident education in terms of operative exposure, self-assessed knowledge, overall rotation experience, and academic productivity.^10–14^ Another way to assess the resident perspectives on becoming competent in thyroid surgery is represented by the survey. However, this method of investigation shows some limitations: firstly, the results are based on self-report by residents; secondly, the concept of competence and the resident's feeling to be competent are highly variable.^1,10,12,15,16^

The purpose of this multicenter study was to analyze the impact on the patient outcomes of total thyroidectomy (TT) performed by residents with close supervision and assistance of attending endocrine surgeons.

## METHODS

Ten European Academic Departments of endocrine surgery participated in this multicenter cohort study: 8 in Italy (Bari, Pisa, Rome, Naples, Rome, Padua, Genoa, and Naples), 1 in France (Marseille), and 1 in UK (London). All of them provided relevant information regarding thyroid surgery performed by a total of 90 surgeons between January 1, 2009 and December 31, 2013. Moreover, the preliminary experience of the participating surgeons at the beginning of the study consisted in at least 10 TT.

Medical records of all patients who underwent TT during the study period were evaluated. Demographic data, preoperative diagnosis, extension of goiter, type of surgical access and surgical approach, operative time, use and duration of drain, length of hospitalization, thyroid histology, and postoperative complications, were recorded. Exclusion criteria were represented by surgery for recurrent disease, lymph node dissection, thyroidectomy plus laryngectomy, subtotal or near-TT, loboisthmectomy, parathyroid autotransplantation, concurrent primary hyperparathyroidism, use of intraoperative neuromonitoring, in order to clear the analysis from a possible susceptibility bias. Moreover, patients treated by surgeons with <10 TT in their experience and cases with missing data or lost to follow-up were not included in the study.

Preoperative evaluation included measurement of thyroid function and autoantibodies, serum calcium, inorganic phosphorus, and magnesium. Ultrasound-color Doppler imaging of the neck with thyroid volume determination, plain chest and neck radiography, and evaluation of vocal cord function through flexible fiberoptic laryngoscopy were always performed before surgery. A multidetector computed tomography scan with multiplanar reformatting and volume-rendering reconstructions of the neck and chest was performed when the goiters seemed to be substernal at ultrasonography, defining cervicomediastinal a goiter with >50% of the gland below the clavicle.^17,18^

Patients were divided into 3 groups: A, patients operated on by an attending surgeon (AS) assisted by a resident; B and C, patients operated on by a junior (postgraduate years [PGY]: 1–3) and a senior (PGY: 4–6) resident surgeon (RS), respectively, both assisted by an AS. AS involved in the study were 10, while RS were 80. All European Academic Departments of endocrine surgery taking part in this multicenter study acted in accordance to the contents promoted by the Accreditation Council for Graduate Medical Education (ACGME) for residents’ training. According to the ACGME—Case Log Coding Guidelines in Otolaryngology, a resident assistant surgeon performs <50% of the TT, or ≥ 50%, but not the key portions of the procedure; an RS performs ≥50% of this one, including the key portions of the procedure, with the AS as supervisor that instructs and assists him.

Written informed consent, also stating that the surgical procedure could be performed by the AS or by an RS assisted by an AS, was always obtained and all TT were conducted using a standardized capsular dissection technique through a collar incision^19^ or through minimally invasive video-assisted thyroidectomy (MIVAT) approach. Thyroid vessels were controlled individually and divided either with conventional knot-tying or with the harmonic or radiofrequency scalpel. In the cases of cervicomediastinal goiters, the TT was performed through a cervical approach, when the thoracic component could be manually retracted to the cervical region, otherwise a manubriotomy was performed. Electrocautery was avoided after the opening of the cervical fascia, and the hemostasis was achieved, close to the recurrent laryngeal nerves (RLNs) or parathyroid glands, by ligatures, bipolar pliers, or biosurgical agents.^20^ The meticulous dissection of RLN and parathyroids was conducted also using loupe magnification.^21^ RLN was systematically searched where it crosses the inferior thyroid artery and traced to the cricoid cartilage. Parathyroid glands where identified and preserved in situ. Drainage was employed in selected cases.

Postoperative clinical evaluation tested for dysphonia, dyspnea, dysphagia, paresthesia, facial muscle and carpopedal spasm, irritability, weakness, and cardiac arrhythmias. Flexible fiberoptic laryngoscopy was performed in patients with hypofunctioning vocal cords at extubation or with an immediate postoperative dysphonia, dyspnea, or dysphagia, although a possible underestimation of subclinical RLN injury. In the analysis, the hypomotility without paramedian paralysis (paresis) or the absence of motility with paramedian position (paralysis) of the vocal cords were grouped together as RLN palsy. When RLN palsy was identified, the patient was seen in weekly follow-up in association with speech therapy and steroid medication for the first 3 months, and thereafter every 4 weeks until recovery was obtained. Serum calcium levels were routinely evaluated 1 day before surgery, daily during the 1st and 2nd postoperative days, then 2 times during the 1st week, and weekly until 1 month. If levels of serum calcium were <10% of preoperative levels in asymptomatic patients, or when symptomatic hypocalcemia was evident, oral calcium carbonate (1–6 g/d) was administered. A minimum of 6 months follow-up period was routinely adopted to confirm any definitive RLN palsy or permanent hypoparathyroidism.

Between-group comparisons were made using Student *t* test for independent samples, and frequencies were compared by χ^2^ test. A univariate logistic regression reporting odds ratios (ORs) and 95% confidence intervals (CIs) was performed to estimate the association of postoperative complications and groups. A multivariate logistic regression was performed to adjust ORs for age, sex, preoperative diagnosis, goiter, surgical access, and approach. A Bonferroni-corrected *P* < 0.01 was considered statistically significant. The analyses reported were performed with Stata 12 (StataCorp LSJ, College Station, TX).

## RESULTS

A total of 10,710 patients underwent thyroid surgery during the study period. According to the exclusion criteria, 1789 (17%) patients were not included in the study: recurrent disease (N = 43; 0.4%); lymph node dissection (N = 183; 1.7%); thyroidectomy plus laryngectomy (N = 19; 0.2%); subtotal or near-TT (N = 23; 0.2%); loboisthmectomy (N = 107; 1.0%); parathyroid autotransplantation (N = 254; 2.4%); primary hyperparathyroidism (N = 38; 0.4%); intraoperative neuromonitoring (N = 1039; 9.7%); operation performed by surgeons with <10 TT in their experience (N = 83; 0.8%). In addition, 13 (0.1%) patients were not included because of missing data or lost to follow-up. The study population consisted of 8908 patients (mean age of 51.1 ± 13.6 years, range: 10–93), of which 6602 (74.1%) were females. Table 1 shows the demographic data and preoperative diagnosis. AS have operated on 7092 (79.6%) patients (Group A), the patients operated on by the overall RS were 1816 (20.3%), of which 261 (2.9%) by the junior residents (Group B) and 1555 (17.5%) by the senior ones (Group C). Each AS and RS performed an average of 709.2 and 22.7 TT, respectively, during the whole study period, and these values were similar in each center. No significant difference in age and sex could be observed among the 3 groups. Preoperative malignancy was detected by fine-needle aspiration biopsy in 1087 (12.2%) and hyperthyroidism in 1429 cases (16.0%). Malignancy was significantly (*P* < 0.001) more common in B (23.3%) and in C (15.8%) vs A (11.0%), as well as in B vs C (*P* = 0.002).

**TABLE 1:**
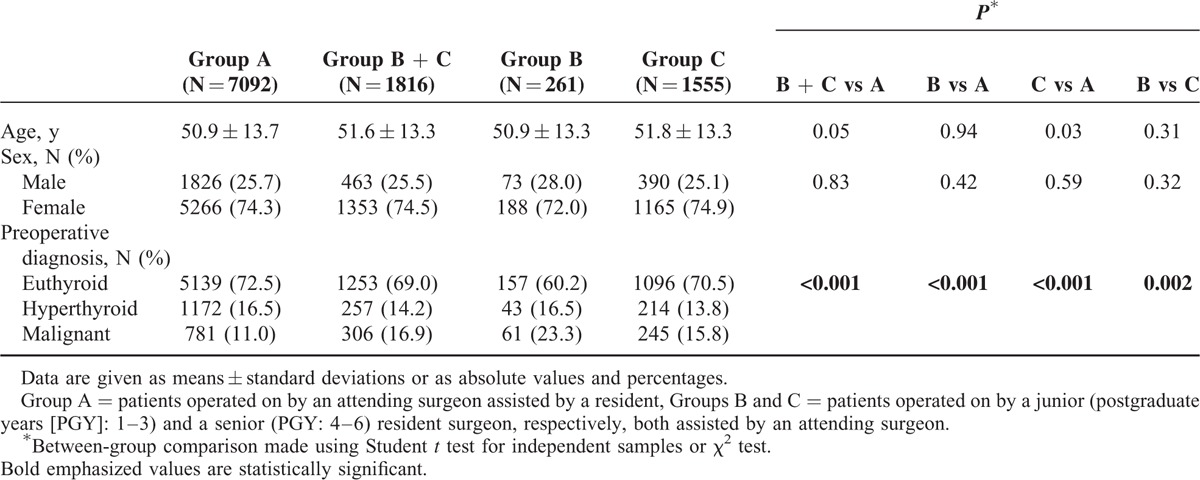
Demographic Data and Preoperative Diagnosis

Data concerning extension of goiter, type of surgical approach, and technique are summarized in Table 2. In 1005 patients (11.3%), the goiter was substernal and a manubriotomy was performed in 8 cases (0.11%). Cervicomediastinal goiters were significantly (*P* < 0.001) more common in Group C vs B (13.2% vs 4.2%) and in Group A vs B (11.1% vs 4.2%). MIVAT was adopted in 1432 cases (16.1%), being significantly more commonly employed in Group A vs B (17.4% vs 5.7%), and C (11.4%; *P* < 0.001), and in Group C vs B (*P* = 0.006).

**TABLE 2:**
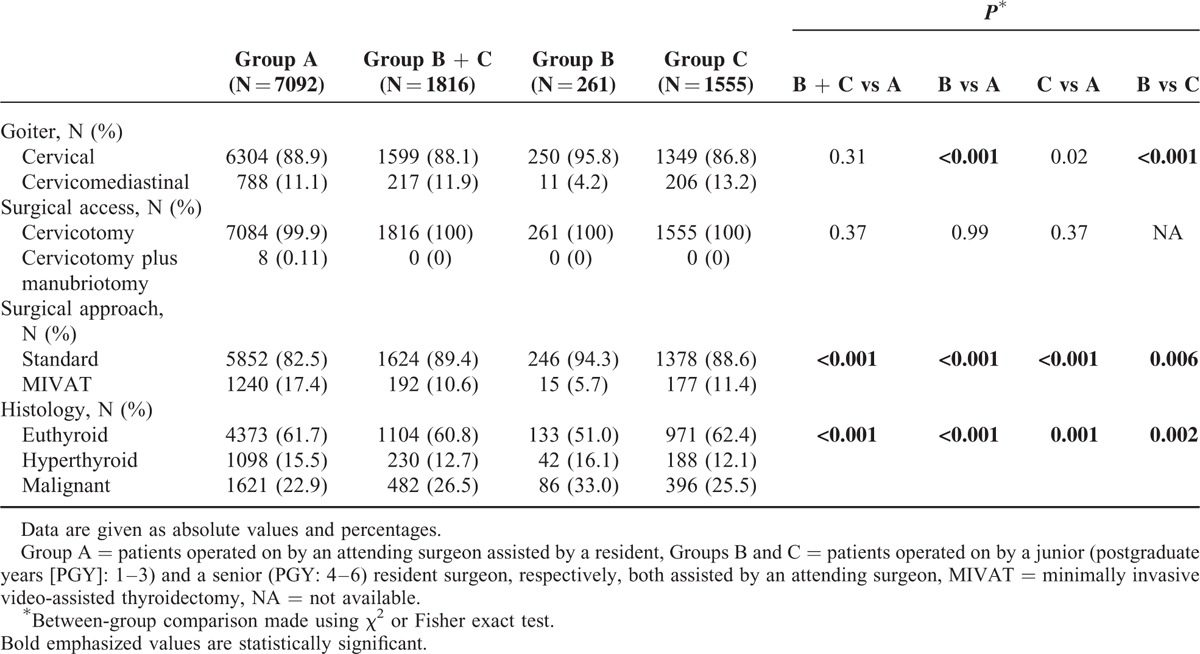
Extension of Goiter, Surgical Access and Approach, and Histological Findings

Operative time was significantly greater (all *P* < 0.001) in Group B (101.3 ± 43.0 min) compared with A (71.8 ± 27.6 min) and C (81.2 ± 29.9 min). Drain was employed in 8068 patients (90.6%), and it was significantly more common in Group A (6369 patients, 89.8%) vs B (219, 83.9%; *P* = 0.002) and vs C (1480 patients, 95.2%; *P* < 0.001) and in C vs B (*P* < 0.001). Duration of drain was significantly lower (all *P* < 0.001) in Group A (47.4 ± 13.2 h) vs C (56.4 ± 16.5 h), and in B (42.8 ± 14.9 h) vs A and C. Length of hospitalization was significantly longer (all *P* < 0.001) in C (3.8 ± 1.8 days) vs B (2.4 ± 1.0 days) and A (2.6 ± 1.5 days).

At histology (Table 2), malignancy was confirmed in 2103 patients (23.6%) and was significantly more common in B vs A (33.0% vs 22.9%, *P* < 0.001) and C (25.5%, *P* = 0.002), as well as in C vs A (*P* = 0.001).

No mortality occurred. Overall postoperative morbidity was 22.3%, and Table 3 shows detailed postoperative complications, compared among the 3 groups using univariate logistic regression. Although no significant difference was observed in Group A (22.3%) vs B + C (22.5%) in terms of overall morbidity, it was significantly higher in B vs A (29.5% vs 22.3%; OR 1.46, 95% CI 1.11–1.92, *P* = 0.006) and vs C (21.3%; OR 1.55, 95% CI 1.15–2.07, *P* = 0.003). No differences were found for all kinds of RLN palsy and hypoparathyroidism, hemorrhage, wound infection, and the “others” among the groups. A higher seroma prevalence was observed in B vs A (1.9% vs 0.1%; OR 17.27, 95% CI 4.41–60.36, *P* < 0.001) and vs C (0.2%; OR 10.08, 95% CI 1.95–65.32, *P* = 0.005).

**TABLE 3:**
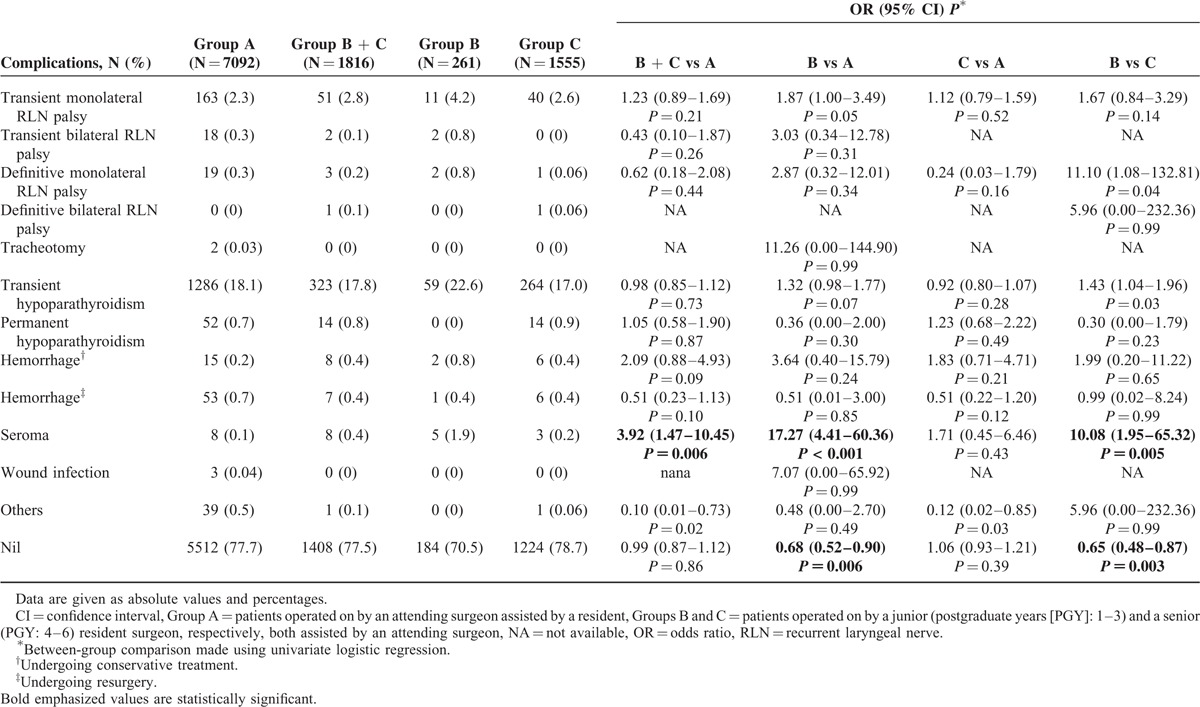
Postoperative Complications: Univariate Analysis

At multivariate logistic regression, adjusting ORs for age, sex, preoperative diagnosis, extension of goiter, type of surgical access, and approach, most of the results of the univariate analysis were confirmed (Table 4). The adjusted ORs for overall morbidity showed a significant association with Group B vs A (OR 1.48, 95% CI 1.12–1.96, *P* = 0.005) and vs C (OR 1.60, 95% CI 1.19–2.17, *P* = 0.002), while no difference was observed in Group A vs B + C.

**TABLE 4:**
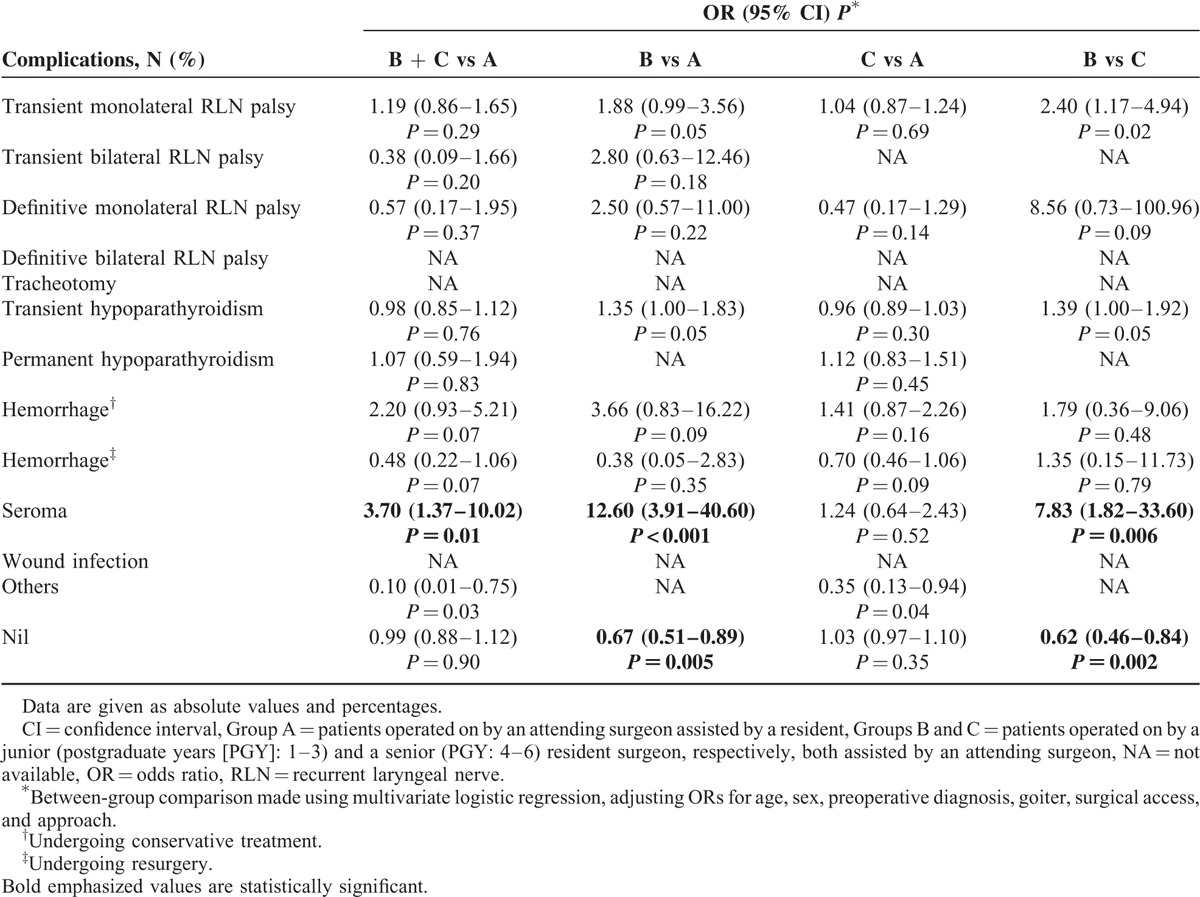
Postoperative Complications: Multivariate Analysis

## DISCUSSION

It is believed that the professional expertise greatly affects on the success of the surgery; therefore, the surgical skill derives from appropriate training and evolves from a continuous practice within a specialty. Several studies support the relationship between surgeon volume and outcomes also in thyroid surgery.^5,7–9^ Regrettably, only few studies analyzed the impact of primary involvement of residents in training in thyroid surgery on the patient outcome.^6,22–24^ The explanation could be that there is a longstanding conflict between the need of medical education, requiring training for inexperienced surgeons, and the priority of patient safety, that should be guaranteed by the most experienced available surgeons.

The results of this study should be interpreted on the basis of its retrospective nature and of the bias due to the significant differences in size and in many parameters of the 3 groups; moreover, the multicenter design could affect the uniformity of diagnostic and therapeutic methods. The statistical analysis was weak and further studies are needed to reach more reliable conclusions. However, no previous studies have been designed like this in literature.

The present study aims to improve the knowledge in this area by analyzing the results obtained in teaching hospitals on a large series of patients, comparing all variables between the cases operated on by AS and RS, subgrouped in PGY 1 to 3 and PGY 4 to 6. In this study, the RS assisted by an AS performed 20.4% of TT. Similarly to previous reports,^6,22–24^ no significant differences in age and gender distribution between patients operated on by AS and by RS were observed. In contrast to the reported literature,^6,22–24^ the AS have significantly managed more cases of thyrotoxicosis; the explanation could probably be linked to the surgical complexity correlated to the hypervascularization of the gland. Otherwise, the preoperative cases of malignancy have been treated more frequently by RS, and especially by junior ones, probably because the neoplasms were preoperatively detected into a single nodule of a normal or almost normal thyroid at ultrasonography, assuming an easier surgery. Indeed, the association of histological malignancy with thyroid disease was significantly higher in the RS groups (27.0%) in comparison with the AS one (23.0%), and in the junior RS group (33.0%) relative to the seniors’ one (25.0%).

The distribution of cervical and cervicomediastinal goiters did not show differences between the AS and the overall RS but, as expected, the junior RS have performed statistically more frequently TT for cervical goiters. Although no significant differences could be observed regarding the type of surgical access among the groups, the senior RS have performed statistically more frequently TT for cervicomediastinal goiter, because of the owned greater surgical skill. Presumably the inherent difficulties in the treatment of the cervicomediastinal goiter explains the precautionary choice by AS to not entrust the cases to the junior residents, but to perform the operation firsthand or assisting the seniors. However, as previously reported,^25^ these data confirm that the cervical approach for the treatment of the cervicomediastinal goiters is feasible in the most cases.

The same rationale seems to clarify the data regarding the used surgical approach; if the standard approach was statistically more frequently adopted by each group of residents, the MIVAT one seems to be a prerogative of the AS; moreover, the tendency toward MIVAT was detected in the senior RS group, as previously explained.

Operative time, duration of drain, and length of hospitalization significantly increased in TT performed by overall RS compared with the AS group.

Similarly to previous reports,^6,22,25–28^ the overall postoperative morbidity was 22.3%. No statistically significant difference was observed in terms of global outcome between the cases operated on by AS (22.3%) and by the overall RS (22.5%). On the contrary, the overall morbidity was significantly higher in the junior resident group (29.5%) than the seniors’ one (21.3%).

Postoperative hypocalcemia and RLN palsy are the most common complications following thyroid surgery, and their reported incidences are very variable. The transient hypocalcemia, indeed, has been reported between 0.6% and 83%,^29–32^ while the permanent one can reach 32%.^29,33–38^ Besides, transient RLN palsy shows an incidence ranging from 0.5% to 18%, while the definitive is less frequent (0–4%).^33,39–42^ The results of this study demonstrate that the seroma incidence was significantly associated with the overall RS group when compared with the AS one. Moreover, as previously noted,^6,22–24^ no statistical difference was found in terms of RLN palsy, hypoparathyroidism, hemorrhage, and wound infection, confirming that thyroid surgery could be safely performed by residents under close supervision. In addition, these data are very important considering the exclusion of the use of parathyroid autotransplantation and neuromonitoring, recognized methods to improve the outcome.^29,43^ The reason of these exclusions was that these 2 precautions were not homogeneously employed in all the participating centers and during all the study period.

On the basis of other experiences,^25,44–49^ age, sex, preoperative diagnosis, extension of goiter, surgical access, and approach have been selected in the multivariate analysis to estimate the association of overall morbidity and complications with the 3 groups. However, the study showed that the overall morbidity was significantly associated with the surgical skill of RS, independently from the effects of the other chosen variables. Specifically, a significantly higher overall postoperative morbidity was found in the junior RS group in comparison with the AS group and with the senior RS one, whereas no difference was evident comparing the AS group with the whole RS one.

In conclusion, this study confirms the importance of an amplified training in thyroid surgery, being increased the need of endocrine surgeons during the last years.^1,10^ Morbidity persists within an acceptable range, even if TT is performed by a surgeon-in-training directly attended by a supervisor. This is of paramount importance given the need to train new endocrine surgeons, but it underscores that a lower level of training influences the overall complications rate. Indeed, the subgroup analysis of junior residents shows that this operation should be always approached as a major surgery procedure; when a young surgeon performs a TT, the risk of complication could be higher, and it should be preserved by a more careful application from the AS. Moreover, this multicenter study underlines the critical role of surgical educators, who are torn between the task to teach, supervise, and evaluate the RS and to guarantee the better outcome for the patients. This seems to be a worldwide need that is still waiting for a resolutive answer. We suggest that TT can be safely performed by residents with close supervision. Innovative gradual training models for residents, appropriate for the reached level of surgical skill and knowledge in dedicated high-volume hospitals, should be proposed in order to allow adequate autonomy for the RS and safeguard patient outcome.
